# Standing up for representation in undergraduate medical education curricula through medical student, librarian, and faculty collaboration: a case report

**DOI:** 10.5195/jmla.2024.1939

**Published:** 2024-10-01

**Authors:** Ellen M. Hong, Rami Atoot, Megan E. Decker, Alexander C. Ekwueme, Cairo Stanislaus, Tadé Ayeni, Christopher P. Duffy, Allison E. Piazza, Mariela Mitre, Linda D. Siracusa, Jennifer F Zepf

**Affiliations:** 1 ellen.hong@hmhn.org, Medical Student, Hackensack Meridian School of Medicine, Nutley, NJ; 2 Medical Resident, Englewood Health, Englewood, NJ; 3 megan.decker@hmhn.org, Medical Resident, Jersey Shore University Medical Center, Neptune City, NJ; 4 alexander.ekwueme@hmhn.org, Medical Student, Hackensack Meridian School of Medicine, Nutley, NJ; 5 Medical Resident, Department of Urology, UMass Chan Medical School, Worcester, MA; 6 tade.ayeni@wsu.edu, Director of Leadership Education, Assistant Professor, Elson S. Floyd College of Medicine, Washington State University, Spokane, WA; 7 christopher.duffy@hmhn.org, Associate Dean, VP Medical Library Services, Hackensack Meridian School of Medicine, Hackensack Meridian Health, Nutley, NJ; 8 alp7016@med.cornell.edu, Clinical Medical Librarian, Samuel J. Wood Library, Weill Cornell Medicine, New York, NY; 9 mariela.mitre@hmhn.org, Staff Dermatologist, Department of Internal Medicine, Hackensack University Medical Center, Hackensack, NJ, and Assistant Professor, Hackensack Meridian School of Medicine, Nutley, NJ; 10 linda.siracusa@hmhn.org, Professor, Department of Medical Sciences, Hackensack Meridian School of Medicine, Nutley, NJ; 11 jennifer.zepf@hmhn.org, Associate Professor, Department of Medical Sciences, Hackensack Meridian School of Medicine, Nutley, NJ

**Keywords:** Medical Education, diverse skin images, Diversity, equity, and inclusion, clinical diagnostic reasoning, reducing healthcare disparities

## Abstract

**Background:**

A shortage of images of pathology on diverse skin tones has been recognized for decades in health professions education. Identifying skin manifestations of disease depends on pattern recognition, which is difficult without visual examples. Lack of familiarity with visual diagnosis on skin of color can lead to delayed or missed diagnoses with increased morbidity and mortality. As the United States continues to increase in ethnic and racial diversity, addressing the disparity in health outcomes with education is vital.

**Case Presentation:**

At the Hackensack Meridian School of Medicine, students, librarians, and faculty came together to address this problem and develop a database of dermatological conditions in people with darker skin tones. A student group initiated a series of meetings with faculty to determine the best approach to address and enhance the representation of diversity in disease images within the curriculum. With the guidance of faculty and librarians, students performed a literature search and created a database of images of skin pathologies in people with darker skin tones. The database was disseminated to course directors and lecturers, and the noted disparities were corrected for the next cohort of students. The database provides an easily accessible resource for creating lecture slides.

**Conclusion:**

This project brought awareness of the need for inclusivity and generated a broad review of the curriculum to be more representative of all patient populations. Most importantly, our experience provides a roadmap for institutional change through student, librarian and faculty collaboration and cultivation of a culture of optimism and acceptance.

## BACKGROUND

Efforts within medical education to address the health inequities laid bare in Unequal Treatment have focused on specific interventions such as improving instruction of the social determinants of health, implicit bias, and upstander training [1,2]. An area sometimes overlooked during curriculum revisions are the curricular materials themselves, which present a critical opportunity to mitigate implicit bias in US physician graduates [3]. Presenting pathology on diverse skin tones and properly contextualizing racial associations of disease is essential early in medical school curricula to engender the knowledge, skills, and attitudes necessary for the equitable practice of medicine.

Improvements in the images available for educational use are urgently needed, as diversity in medical images throughout the medical literature is an ongoing issue. Lester et al. (2019) described a need for a commitment to documenting, photographing, and publishing manifestations of disease in a wide variety of skin tones [4]. This disparity was also evident during the height of the COVID-19 pandemic, with a lack of published images of COVID-19 cutaneous manifestations in patients of color, which may have contributed to poorer outcomes [5]. Clearly, the ability to identify novel etiologies of disease and evolving threats depends on inclusive literature and timely dissemination of knowledge of presentations in patients with darker skin tones.

This lack of diverse imagery, especially in dermatology textbooks, is significant because differences in skin tones can affect how diseases manifest [6]. In dermatologic textbooks, certain presentations are described as “classic,” such as redness [4,5,7]. These presentations can be missed in patients of color, as more pigment in the skin can mask redness, obscuring the “classic” signs. For example, Raynaud's disease is frequently described as “red, white, and blue” discoloration of the digits, a description that necessitates a background of fair skin to enable detection. Approximately 3–5% of the general population suffer from Raynaud's disease [8]. It is important to identify it in patients with darker skin tones because it can be a sign of an underlying connective tissue disorder, and these conditions impact Black patient populations more severely. For example, the prevalence of systemic lupus erythematosus is four times higher in people who are Black than White [9]. In addition, Black and Native Americans with systemic sclerosis tend to have more severe disease phenotypes than White Americans [10].

Equally worrisome is the consequence of missing the diagnosis of skin cancer in patients with darker skin. Unfamiliarity with the presentation of skin cancers such as basal cell carcinoma, squamous cell carcinoma, and melanoma in people with darker skin can lead to diagnosis of the cancer at later stages, delay the time to treatment, and result in poorer outcomes [11]. For example, in a study of trends in melanoma mortality, White patients had the highest incidence of the disease, yet patients who were non-White suffered the highest mortality [12]. A recent scoping review found evidence suggesting that “socioeconomic factors, lack of access to healthcare, the presence of bias, and deficient skin cancer education among non-White populations as well as lack of physician training may contribute to the disparity in mortality rates related to melanoma in this group” [13]. These findings highlight the need to expand the teaching of health disparities to encompass the social determinants of health as well as to identify additional factors underlying such disparities in order to develop effective solutions.

## CASES PRESENTATION

The pre-clerkship phase at Hackensack Meridian School of Medicine (HMSOM) is a clinically framed and deeply integrated curriculum spanning 16 months [14]. Relevant pathologies are presented throughout seven courses: Molecular and Cellular Principles, Structural Principles, Infection Immunity and Cancer, The Developing Human, Homeostasis and Allostasis, Nutrition Metabolism and Digestion, and Neurosciences and Behavior. Based on adult learning theory, evidence-based active pedagogical approaches tied to session learning objectives are utilized within every course to promote the achievement of session content and course learning objectives. Large-group sessions utilize slides to present basic biomedical science foundations and clinical presentations of disease. Throughout the session, students meet in pairs and/or small groups to evaluate case vignettes, discuss related questions, and then report their findings back to the entire class.

In July 2018, the inaugural cohort began the Phase 1 pre-clerkship curriculum. As part of the HMSOM's continuous quality assurance process, student and faculty feedback led to improvements in the initial curricular offerings. Our 2020 cohort entered the HMSOM at the same time as the US was reckoning with the racially motivated murders of Ahmaud Arbery, Breonna Taylor, and George Floyd amid revelations of pervasive violence against people of color by law enforcement and its institutions. This prompted urgent discussions about race across academic institutions; at our medical school, discussions around structural racism and its impact on health outcomes were held in a variety of forums and included evaluating the curriculum to address this ongoing public health crisis [15].

While curriculum leaders were planning this work, a student of color found that his skin tone was not represented in the images of skin pathologies presented during dermatology sessions. A group of like-minded students came together after realizing this was a recurring gap in the curriculum. They hoped to bring about positive change to improve subsequent versions of the course and enhance integration across all segments of the Phase 1 curriculum. The students sought guidance from faculty and staff at the HMSOM, leading to the creation of a student-led task force that included the expertise of course directors, medical librarians, and the director of the Office of Diversity, Equity and Inclusion.

The joint student and faculty task force had multiple discussions which mobilized around several priorities: 1) creation of an online library resource and diverse skin tone images database for faculty teaching in Phase 1, 2) communication of concerns to student representatives on curricular committees, and 3) researching the possibility of a network-wide image repository program.

The first and most urgent priority was the creation of a library resource and diverse skin tone images database. This resource would be made immediately available to course directors and teaching faculty in need of images to create comprehensive teaching materials and course content that fully represented the diversity of clinical presentations of dermatological diseases across the human population. Medical librarians located several dermatology textbooks, and finding gaps in our collection, purchased additional textbooks for this mission. Medical librarians then created an online Toolkit, using LibGuides by Springshare, called “Representation in Medicine,” which listed these texts and other resources [16]. With a broad understanding of representation, we also used this opportunity to include texts on cultural competence, LGBTQIA+ health, and medical racism.

Students then generated a list of diseases they wanted to see more diversely represented in session materials, focusing on infectious and inflammatory conditions. Conditions of high frequency (e.g., eczema) and high impact (e.g., toxic epidermal necrolysis) were prioritized. Non-dermatologic manifestations of disease (for example: jaundice, hyperpigmentation, and petechiae) will be added over time.

Beginning in April 2021 and with the guidance of medical librarians, students searched the textbook literature and PubMed for images using keywords for specific pathologies. This search resulted in 28 source publications for 88 images of 57 conditions. The images, in JPEG format, were organized alphabetically by condition and hosted on a new LibGuide (Springshare), which we titled “Diverse Skin Images Database.” The complete citations for each image were also provided, with a direct link to the source material so the image could be viewed in context. Since many of the images were collected from proprietary textbooks, copyright rules dictated that we password-protect the Database. The password was then broadly shared with students, staff, and faculty at the HMSOM. At the same time, the librarians and faculty vetted a trial of Visual Dx(tm), a subscription-based image library featuring clinical images of disease in diverse skin tones, and this resource was eventually acquired [17].

Faculty and librarians then curated a collection of related open access databases and resources for posting within the Toolkit alongside the database. This Toolkit includes several reviews, articles, opinion pieces, books, and other resources that have been published to address the misrepresentation of race and gaps in diverse imagery. One such resource comes from medical student Malone Mukwende, co-author of *Mind the Gap* [18,19]*.* The purpose of this clinical handbook is to serve and help healthcare professionals learn how diseases can manifest in patients with darker skin. The Toolkit currently contains *Mind the Gap* along with textbooks specializing in images of disease on skin of color and databases of high-quality open access images of medical conditions in a range of skin tones (Table 1).

**Table 1:** Resources within the Representation in Medicine Toolkit at Hackensack Meridian School of Medicine

Textbooks specializing in images of disease on skin of color^a^	Online resources containing high-quality open-access images of medical conditions on a range of skin tones
Alexis AF, Barbosa VH. *Skin of Color: A Practical Guide to Dermatologic Diagnosis and Treatment*. Springer; 2013.	Black … brown skin https://www.blackandbrownskin.co.uk/
Donkor CMYA, Aryee-Boi J, Osazuwa IR, Afflu FK, Alexis AF. *Atlas of Dermatological Conditions in Populations of African Ancestry*. Springer; 2021.	University of New Mexico's Inclusive Dermatology: Creating a Diverse Visual Atlas of Skin Conditions http://hsc.unm.edu/medicine/departments/dermatolog
Jackson-Richards D, Pandya AG. *Dermatology Atlas for Skin of Color*. Springer; 2014.	Skin Deep: A DFTB Project https://dftbskindeep.com
Love PB, Kundu RV, eds. *Clinical Cases in Skin of Color: Medical, Oncological and Hair Disorders, and Cosmetic Dermatology*. Springer; 2016.	American Academy of Dermatology Association Skin of Color Curriculum https://www.aad.org/member/publications/impact/2022-issue-3/new-skin-of-color-curriculum-is-here
Moiin A, ed. *Atlas of Black Skin*. Springer; 2020.	Cutis - The Latest in Skin of Color https://www.mdedge.com/dermatology/skin-color
Taylor SC, Kelly AP, Lim HW, Anido Serrano AM, eds. *Taylor and Kelly's Dermatology for Skin of Color*. Second edition. McGraw-Hill Education; 2016.	

a Additional textbooks dating back to 1981 can be found at: https://skinofcolorsociety.org/search-results/uncategorised/skin-of-color-dermatology-textbooks

The student-led task force and database represents a just-in-time intervention for on-the-ground deployment for rapid utilization. We found that course directors and faculty were immediately amenable to using a database to create course and session materials. The newly created and evolving curriculum presented a relatively malleable substrate for this intervention. For faculty accustomed to teaching with specific slides, the Database and Toolkit are easily searchable resources for replacement and/or addition of images.

The Database and Toolkit have been available on the library homepage since February 2021 and are maintained and updated as additional images and resources become available [16]. Before every Phase 1 course, faculty and course directors are advised of this resource and encouraged to use it for their course and session revisions. Since its launch, most course directors have accessed the database for course materials. Since their creation in 2021, the Toolkit has been viewed 1,954 times and the Database has been viewed 542 times [20].

Students have noted how much they appreciate when faculty include images of diseases in patients with darker skin tones. “Students have shared that the resources have been invaluable in enhancing their understanding and diagnosis of dermatologic diseases in people of color,” says HMSOM medical student, Alexander C. Ekwueme (email communication, May 23, 2024). “This progress underscores the crucial need for inclusive medical education, providing future healthcare professionals with the knowledge and tools to deliver equitable care. The positive feedback highlights the Toolkit's role in filling a vital gap in medical training and its potential to improve clinical outcomes for diverse populations.”

## DISCUSSION

Given the array of diseases that manifest in skin, healthcare professionals across all specialties will encounter patients with signs and/or symptoms related to the skin. Educational initiatives to increase awareness, knowledge, and diagnostic skills to enable accurate disease detection is imperative to ensuring that every patient receives the highest quality of care, regardless of skin tone. Recognizing the unique opportunity and privilege of serving patients in one of the most diverse states in the country, we aim to utilize the broad reach of our parent health network to establish procedures for the submission of diverse presentations of pathology seen in clinics and hospitals throughout the network. Licensing and copyright restrictions prevent us from publicly sharing all the images currently in the Database, but over time, we hope to utilize many more open-source images to make this database a public resource with the potential of accepting submissions to benefit all healthcare students and professionals worldwide.

These joint efforts to enhance and increase the teaching of skin diseases in people of color were endorsed and embraced by the Dean of HMSOM, Jeffrey R. Boscamp. As the teacher of a session entitled “Dermatologic Manifestations of Infectious Skin Diseases,” he incorporated photographs from the Representation in Medicine Toolkit into his slides and described different skin disease presentations in patients with a broad spectrum of skin colors. His example set a high bar for faculty at the HMSOM as well as across academic institutions to follow.

Our students, librarians, and faculty continue to screen the literature for additional open access resources and plans to expand the Database are underway. The work of this group led to recognition by the HMSOM Medical Education Committee (MEC), which in July 2022 established the Cultural Humility Curriculum Subcommittee. This group is charged with providing the MEC with guidance on the development and review of an integrated cultural humility curriculum that builds students’ abilities to care effectively for all patients and populations, with the goal of achieving equitable health outcomes. Since its inception, this subcommittee has audited the Phase I curriculum using the Association of American Medical Colleges Tool for Assessing Cultural Competence Training [21]. Most recently, a recognized thought leader Hetty Cunningham, MD (Columbia University Vagelos College of Physicians and Surgeons) was invited to the HMSOM to support and consult on developing inclusive and anti-bias materials for medical educators [22].

Many influential medical professional organizations, such as the Association of American Medical Colleges (AAMC) and the International Skin of Color Society (SOCS), are working to reverse the effects of structural inequities in the healthcare system. For example, the Arnold P. Gold Foundation seeks to advance humanism in healthcare through compassion, respect and inclusivity of the cultural and ethnic backgrounds of others [23]. The observation of a gap in diverse images, initial discussions around this problem, and the design of the Database were featured at the Gold Foundation Humanism and Honor Society's Structural Racism Initiative in May of 2021. Student and faculty members of our team were invited to share a talk followed by a “Question & Answer” discussion session. Since this presentation, our medical students have continued to add images to the Database and advocate for this initiative throughout our institution as a solution to address healthcare disparities.

Since our initiative to increase the diversity of diagnostic images in dermatology began at our medical school, we discovered that several academic institutions in the US were moving in similar directions to expand their curriculum to include presentations of dermatologic diseases on people of color. Although the depth and breadth of images and resource links vary between schools, these efforts have provided a more inclusive perspective on how dermatologic disease varies among people with different skin colors worldwide. To accomplish this goal, we searched the US for school of medicine websites and listed these databases in Table 2A and Table 2B. The sites in Table 2A house collections of photographs of different dermatologic diseases on people with skin of color. The sites in Table 2B provide substantial resource lists for those wishing to expand their curriculums. These resources include books, videos, and resources housed by private, commercial, and government organizations. We hope that the resources provided in Tables Table 2A and Table 2B will collectively lead to the inclusion of these images and resources in didactic curriculums and expand the knowledge base of trainees across the healthcare professions.

**Table 2A:** Individual SOM Diversity Image Databases

Academic Institution	Name of Database	URL
University of Iowa Health Care	Dermatology Skin of Color Image Atlas	https://www.healthcare.uiowa.edu/skinofcolor/atlas/
University of New Mexico School of Medicine	Photo Gallery of Skin Conditions	https://hsc.unm.edu/medicine/departments/dermatology/inclusive-dermatology/gallery.html
University of North Carolina at Chapel Hill School of Medicine	Dermatology Image Library	https://webapps.med.unc.edu/dil/
Wayne State University School of Medicine	Dermatology Image Atlas	https://medtech.med.wayne.edu/dermatology/

**Table 2B:** University Websites with Collections of Multiple Resources

Academic Institution	Name of Database	URL
Hackensack Meridian School of Medicine	Representation in Medicine	https://library.hmsom.edu/representation
Loyola University Chicago	Loyola University Dermatology Medical Education Website	https://www.meddean.luc.edu/lumen/meded/medicine/dermatology/melton/title.htm
The State University of New York at Buffalo	Skin of Color Resources	https://research.lib.buffalo.edu/skin-of-color-resources
University of South Carolina School of Medicine Columbia	Skin of Color Resources	https://uscmed.sc.libguides.com/medicalimages
Washington University School of Medicine in St. Louis	Brown Skin Matters	https://becker.wustl.edu/news/brown-skin-matters/

Our project focuses on clinical images of disease, however the issue of lack of diversity in representation of disease in medical education extends to illustrations used to provide visual depictions of anatomy, physiology, and pathology. A 2018 study found that only 4.5% of images in commonly required anatomy and physical diagnosis textbooks depicted illustrations including dark skin [24]. Medical Illustrator and Medical Student Chidiebere Ibe brought a spotlight to this issue specifically with his depictions of a range of human conditions, most famously a pregnant black woman with her unborn fetus [25]. This work has led to further initiatives championed by the Association of Medical Illustrators to create equity in medical illustrations for print and digital teaching materials.

We urge educators across the health professions to critically evaluate the images used to depict disease in teaching materials to ensure they are representative of all the patient populations. Furthermore, health science educators should encourage collaborators, authors, and publishers to continue to expand the diversity within the available images of manifestations of disease. By establishing a committee such as the Cultural Humility Curriculum Subcommittee, academic institutions can ensure continuous and sustainable change that encourages partnership of students, faculty, and staff. The benefit of this approach will extend not only to patients, but also to students in the health professions themselves as trainees who feel their race is under-represented or presented in a biased way can experience isolation and burnout.4 Strategies to reduce healthcare disparities with respect to race, ethnicity and gender in the medical student body are critical initiatives that will become fortified when we broaden our lens of representation to the curricular materials themselves.

## Data Availability

There is no data associated with this article.
